# Trends in Mercury Contamination Distribution among Human and Animal Populations in the Amazon Region

**DOI:** 10.3390/toxics12030204

**Published:** 2024-03-07

**Authors:** Irvin Martoredjo, Lenize Batista Calvão Santos, Jéssica Caroline Evangelista Vilhena, Alex Bruno Lobato Rodrigues, Andréia de Almeida, Carlos José Sousa Passos, Alexandro Cezar Florentino

**Affiliations:** 1 Postgraduate Program in Environmental Sciences (PPGCA/UNIFAP), Federal University of Amapá, Rod. JK, km 02, Macapá 68903-419, Brazil; irvmarto@gmail.com (I.M.); vilhena.jessica@gmail.com (J.C.E.V.); 2 Postgraduate Program in Ecology (PPGECO), Institute of Biological Sciences (ICB), Federal University of Pará (UFPA), Augusto Correia, Number 1, Guamá, Belém 66075-110, Brazil; lenizecalvao@gmail.com; 3 Postgraduate Program in Health Sciences (PPGCS), Federal University of Amapá, Rod. JK, km 02, Macapá 68903-419, Brazil; alex.rodrigues@unifap.br; 4 Faculty UnB at Planaltina (FUP/UnB) Planaltina, University of Brasília, Brasília 73345-010, Brazil; andreiaalmeida.df@gmail.com (A.d.A.); cjpassos@unb.br (C.J.S.P.)

**Keywords:** artisanal and small-scale gold mining (ASGM), human health risks, environmental impact, mining impact, spatial distribution, aquatic ecosystems, riverside communities, ecotoxicology, ecoregion, biosphere, dietary habits

## Abstract

Mercury contamination in the Amazon arising from both natural sources and intensive mining activities in the region is a significant public health concern. This metal is used to separate Au from sediments. Accordingly, this study aimed to assess the impact of mining on mercury contamination in the animal and human populations of the Amazon. This overall objective was pursued through a systematic review of the existing literature to assess the impact of Hg and identify gaps in geographic coverage arising from this assessment. Herein, we employed PECO and PRISMA-ScR protocols to select articles published between 2017 and 2023 based on projected points on a map within the biogeographic boundaries of the Amazon. We found that mercury concentrations increase with trophic levels, reaching high values of 3.7 µg/g in the muscles of predatory fish and 34.9 µg/g in human hair. The mean level of mercury in human hair in the whole (Amazon) region exceeds 6 µg/g, surpassing tolerance levels. Although mining regions show high concentrations of Hg, the highest incidence was observed among populations with fish-based diets. It was concluded that continuous research and monitoring of fish in the region are required in order to accurately assess the risk associated with Hg contamination, especially since fish are the main source of protein in this region.

## 1. Introduction

The vast geographical extension of the Amazon region, covering approximately 7 million km^2^, is characterized by remarkable biological diversity and complex ecosystems, including both aquatic and terrestrial environments [1,2,3]. However, this region has been experiencing increasing pressure owing to the intensified encroachment of human activities. The mining sector is one of the main drivers of this pressure. This increase in mining activities has led to the deterioration of vegetation cover and soil erosion, culminating in the degradation of forest areas [4,5,6]. The Scientific Panel for the Amazon identifies these human activities as the primary catalysts for forest degradation, impacting multiple aspects of the ecosystem [7]. 

In the Amazon, the progression of artisanal and small-scale gold mining (ASGM) is primarily driven by the discovery of new gold deposits and stimulated by the profitability of this activity. Its expansion into indigenous territories, which started during the 1980s, was facilitated by weak environmental law enforcement and intense lobbying activities, leading to various socio-environmental implications [8].

This ongoing trend continues to challenge environmental governance [9,10]. Contemporary illegal mining activities in the Brazilian Amazon have experienced exponential growth, increasing by more than 90% since 2017, leading to major forest loss in 2020 [10,11,12,13,14,15], especially during the COVID-19 pandemic [16].

Furthermore, the exploitation of forest resources contributes substantially to the environmental degradation of the Amazon, with small-scale mining playing a crucial role in countries such as Suriname, Ecuador, Peru, and Guyana [17,18,19,20,21].

Gold prospectors employ rudimentary extraction methods involving the use of mercury to capture dense gold particles through gravitational concentrations of water–ore slurry [22]. With their disregard for efficiency and safety, much of the Hg^0^ used by these miners is discharged in mining tailings, in turn contaminating the surrounding water resources [11,23,24,25,26]. This practice eventually leads to the formation of the most toxic and bioavailable form of Hg, methylmercury (CH3Hg^+^), through the conversion of aqueous Hg^2+^ and Hg^0^ by sulfate-reducing bacteria in anoxic aquatic environments. CH3Hg^+^ then moves further up the freshwater food chain via aquatic plant roots and reaches fish trophic levels, where it bioaccumulates and biomagnifies in higher concentrations in the tissues of top predators. This feeding habit as well as the uptake of aqueous CH3Hg^+^ from fish gills is expected to cause high bioaccumulation in the liver, brain, and muscle, with the latter pathway directly affecting the cardiovascular system [27]. High levels of CH3Hg^+^ affects cellular function, leading to irreversible neurological damage in animals and humans [28,29].

Therefore, the contaminated aquatic food chain poses a significant threat to vulnerable Amazonian populations dependent on fishing for subsistence and nutrition from fish as their primary source of animal protein, as it is also their main gateway to Hg exposure [12,15,26,30,31,32]. CH3Hg^+^ comprises approximately 80% of the total mercury found in human hair, and its presence in the biological matrix indicates chronic mercury exposure. Hair, as the biomarker of Hg, is generally preferred over blood, urine, or other tissues, owing to the ease of collecting samples, its non-intrusive nature, and its ability to record long-term exposure. 

This situation prompted the World Health Organization (WHO) and regional Common Market of the South (MERCOSUR) to publish references on Hg threshold levels for food safety and human exposure. 

On the other hand, the Amazon naturally exhibits high levels of Hg from volcanic sources in the Andes, as evidenced by its presence in forest soil, which is carried to tributaries through erosion and then to rivers where the contaminant is diluted and adheres to sediments [33,34,35,36]. However, mining can hasten the release and mobilization of natural inorganic Hg through deforestation and erosion, as well as elemental Hg introduced for the amalgamation process [37,38]. 

Previous reviews of scientific publications reporting on Hg in the Amazon highlighted a considerable spatial gap outside of Brazil, whereas a particular urgency arises for riverine populations whose livelihood and food source depend on fishing. Also, the few studies reporting the exposure of humans to Hg have largely failed to include Amazonian territories outside of Brazil [39]. Scientometric studies on Hg contamination have revealed a more pronounced focus on the Amazon compared to other Brazilian biomes, emphasizing gold mining, fish exposure to Hg, and contamination by Hg in water bodies and river sediments [40,41]. Additionally, a recent systematic review of the geological characteristics of the Amazon basin highlighted the uneven distribution of dangerous metal enrichment, including mercury, owing to mining activities, as well as a scarcity of data from other countries in the Amazon basin [42].

Moreover, a review of feeding guilds and dietary habits in the Amazon revealed relatively high levels of CH3Hg^+^ in predatory fish compared with their non-predatory counterparts [43]. Therefore, this study aimed to assess the impact of Hg on human and animal populations in the Amazon region and identify any gaps in geographic coverage arising from this assessment. The study area includes the international Amazonian ecological region, a vast tropical forest and watershed extending over nine South American countries: Brazil, Peru, Colombia, Venezuela, Ecuador, Suriname, Guyana, Bolivia, and French Guiana [44].

## 2. Material and Methods

The use of Preferred Reporting Items for Systematic reviews and Meta-Analyses (PRISMA) in cataloging articles ensures that the present systematic review has been documented in a clear and transparent manner and that this study is replicable [45,46]. This scoping review was conducted following PRISMA-ScR (Preferred Reporting Items for Systematic reviews and Meta-Analyses extension for Scoping Reviews). VosViewer^TM^ version 1.6.19.0 (Leiden, The Netherlands) was employed as a preliminary tool to identify trends in the search syntax related to mercury concentrations in the Amazon region [47]. The analysis was conducted using the keywords present in the titles and abstracts of studies that were finally accepted for screening. 

Population, Exposure, Comparator, Outcome (PECO) was used as a framework to delineate the research question and establish search criteria [48]. In the present study, PECO criteria were defined as follows:

- Population: Human and animal population in the Amazon region.
- Exposure: Exposure to mercury (Hg).
- Comparator: Different levels of mercury exposure or comparisons between different species or regions, and the values allowed by health organizations.
- Outcome: Hg contamination in animal tissues and humans relative to diet.

All of these criteria played a role in structuring and conducting the research. To support these PECO criteria, articles included in the review had to contain a quantification of mercury levels and make a clear distinction between human and non-human samples. Additionally, the sampling region of each study had to be within the biogeographic province of the Amazon, as defined by the Amazon Network of Georeferenced Socio-Environmental Information (RAISG). The selected studies presented original research and were not reviews or meta-analyses. This ensured that the research would be relevant and applicable to the population and the environment under study. Table 1 shows the search strategy with Boolean parameters used in full form for advanced options in the entire content of the items in the Web of Science and SCOPUS databases from 2017 to 2023.

All identified records were assessed for relevance based on their titles and abstracts. The full texts of potentially eligible studies were retrieved and assessed for eligibility based on the predefined criteria. Studies that did not measure Hg in animal tissues and/or humans or performed tissue collection outside of the Amazon region were excluded. 

Data were systematically extracted from the selected studies using a standardized form. Each article was identified using a DOI link. The following information was recorded for each study:

- Study Characteristics: DOI, title, and author(s) of the study.
- Population Characteristics: Whether the study focuses on human or non-human species, the scientific nomenclature of the studied species, and their feeding guild.
- Collection Details: Sample collection location (in decimal degrees) and the period during which the samples were collected.
- Mercury Exposure Details: Type of tissue sampled and the total amount of mercury found in these tissues.
- Additional Data: Any mention of indigenous regions, hydroelectric dams, or artisanal and small-scale gold mining (ASGM), as well as details of mercury analysis.

This structured approach to data extraction ensured consistency across all studies included in the review. 

The highest average (mean or median) total mercury (THg) level reported in each study was used to indicate accumulation in the target human or animal species. Additionally, Hg data in animals were categorized into their respective dietary guilds (feeding habits). In the present study, piscivorous fish were included in the carnivorous group. This classification distinguishes them from other piscivorous animal species that feed on fish. 

The reported Hg concentrations were assessed according to United Nations (UN) and WHO guidelines for human consumption, and permitted levels of mercury accumulation in food were established. The limit for non-predatory fish is 0.5 µg/g, and the limit is 1.0 µg/g for predatory fish [49,50]. These limits were also recognized by MERCOSUR, according to resolution RDR No. 42 of 29 August 2013 [51]. 

Furthermore, WHO guidelines for Hg in human hair consider concentrations below 1.8 µg/g to be safe, while the range from 1.8 µg/g to 6 µg/g is considered concerning because 6 µg/g is the indicated tolerance level for cellular functions in the human body [52,53]. The WHO acknowledges that these guidelines are not absolute, and that individual susceptibility and exposure patterns may vary. This means that even mercury levels below any of these thresholds may still pose a risk to human health and lead to adverse health effects. Hair Hg concentrations above 10 µg/g are considered hazardous, and may lead to notable chronic symptoms for mercury poisoning [54].

### 2.1. Geoprocessing

Quantum GIS, version 3.20.3 [55], was used to record significant sampling locations projected within the boundary of the Amazonian ecoregion, which is the layer obtained from RAISG [44]. Each georeferenced record was adjusted to include only sampling locations that had (1) a distinctive average (mean or median) or combined average (mean or median) value of Hg per research article, and (2) significant spatial distance from other sampling locations. In this regard, nearby spatial samples with the same THg value, but within 1°–2° in the WGS84 Coordinate Reference System, were represented as a single point.

To examine spatial disparities in the Amazon study area, the region was categorized based on ecoregion distinctions, as defined by Dinerstein et al. [56,57]. These distinctions allow the grouping of areas into their respective bioregions, including the northern, central, western, and southern regions, as well as the Guiana Shield and Amazon Estuary. To analyze recent mining activities, mining polygons delineated by RAISG [44], which are categorized as active and aimed at gold extraction, were integrated into the global mining footprint dataset provided by Tang and Werner [58]. 

The distance between sampling points (animal or human surveys) and mining activity was evaluated using three classes: <20 km, 20 km to 50 km, and >50 km. Up to 20 km, mining directly influences both the sediment and the food chain in the surrounding areas [59,60], while significant Hg contamination is observed at a distance of 50 km, impacting both the local ecosystem and the human population dependent on it for sustenance [61,62]. To accurately measure the proximity of human or animal locations to these high-risk zones, mining area polygons were converted into points. This allowed us to calculate the distance to the edge of the mining areas, providing a more precise measure when compared to the use of centroids of the mining polygons.

### 2.2. Statistical Analysis 

Tabulated mercury data are presented as their mean and median values. The General Linear Model (GLM) was utilized to find statistical relationships among specific tissue Hg concentrations, children, reproductive age, riverside communities, and mining, as well as assess the variation in metal concentration among demographic groups. Logistic regression analysis was also conducted beyond the GLM, transforming total mercury concentration (Hg) into a binomial variable. This transformation was based on an established threshold of 6 μg/g [52,53]. The Akaike Information Criterion (AIC) was used to select the best-fit model. All analyses were conducted using R software version 4.3.1 [63].

## 3. Results

**Figure 1:**
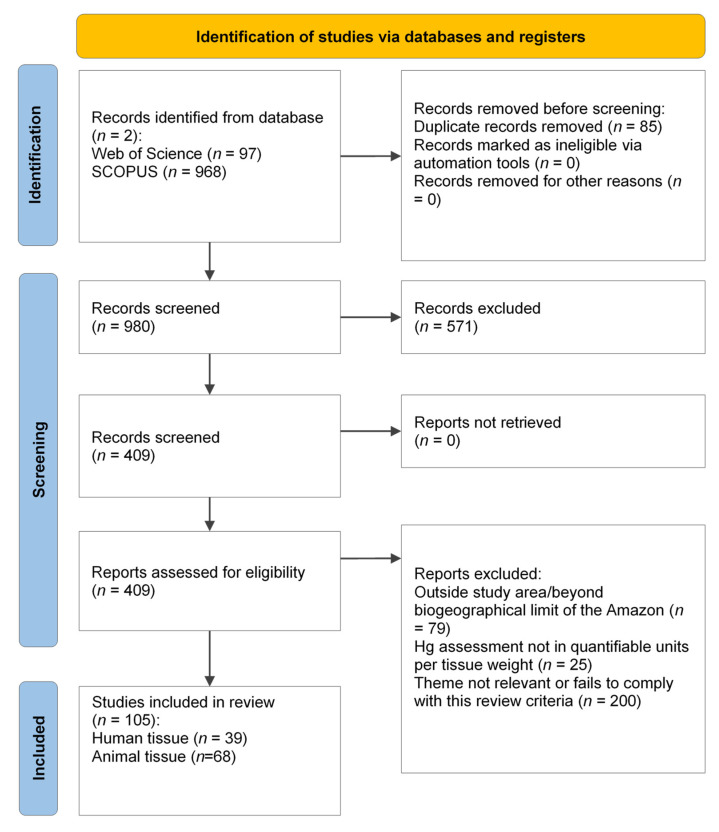
Flowchart of the literature search according to PRISMA 2020 guidelines.

Bibliographic research conducted using the PRISMA 2020 flowchart is shown in Figure 1. Duplicate items were removed before the screening process, which involved reviewing the titles and abstracts of records for relevance to the research theme, followed by analysis of the full text for eligibility criteria. Among the 105 articles accepted after screening, 66 were studies on animals, 37 were solely on humans, and 2 articles contained both animal and human samples.

### 3.1. Study Characteristics

**Figure 2:**
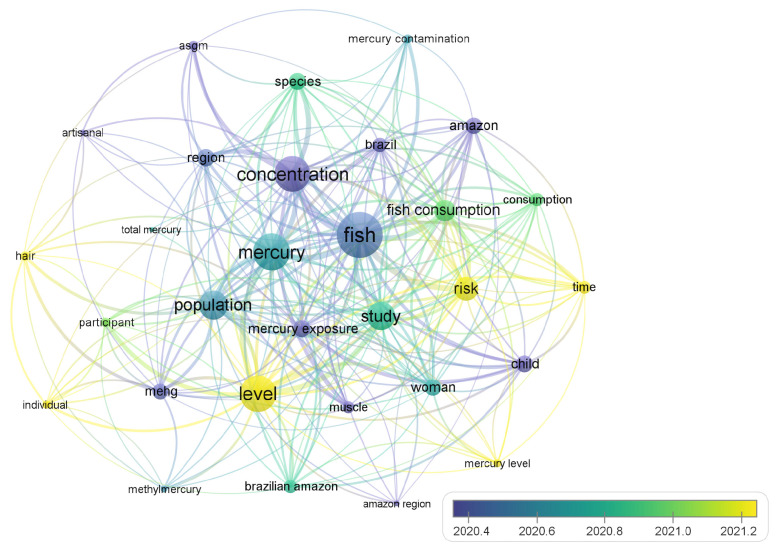
VosViewer keyword analysis with relevance in the year of article acceptance by color coding.

The 105 reports selected through PRISMA were processed using VosViewer to generate 1383 keywords based on the titles and abstracts of the articles. This bibliometric visualization was based on the density of eight occurrences of related keywords. Figure 2 presents a network of keyword co-occurrences in the articles included in the review.

**Figure 3:**
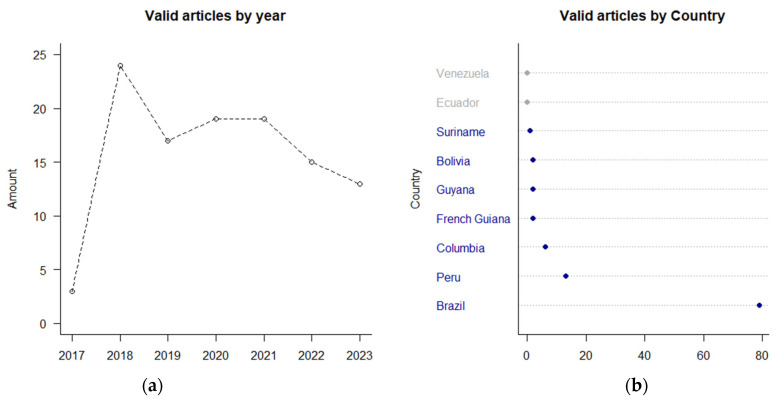
Article occurrences: (**a**) valid for each year; (**b**) valid for each Amazonian country (those not represented are grayed out).

The results indicate a predominance of studies reporting on fish muscle tissues contaminated by Hg caused by artisanal small-scale gold mining (ASGM) (the only pollution activity mentioned), presenting risk factors through fish consumption, and studies predominantly carried out in the Brazilian Amazon region. Additionally, total Hg and methylmercury are measured in human hair and fish muscle tissues as well as the reproductive population. Moreover, the color classification in Figure 2 shows the highest occurrence of relevant keywords in studies between 2020 and 2021, with the largest single volume of studies published in 2018. Figure 3 shows the distribution of the number of articles published on this topic by year and country.

**Figure 4:**
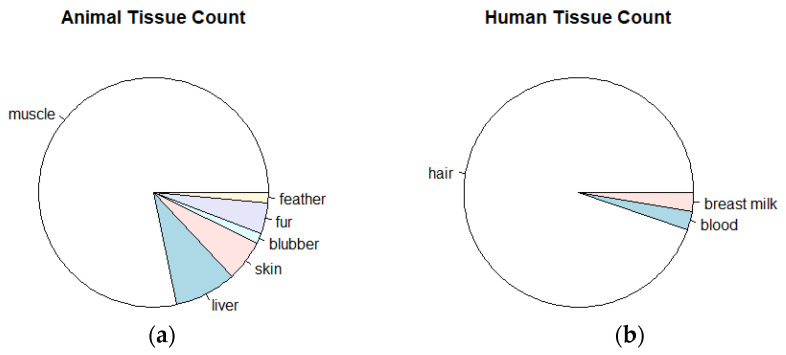
Predominant tissue samples in datasets: (**a**) non-human and (**b**) human, after screening.

Brazil is the most covered country by relevant research about mercury contamination in the Amazon, with a total of 25 published articles. Peru and Colombia recorded 13 and 6 articles, respectively; French Guiana, Guyana, and Bolivia have 2 articles each; and Suriname has 1 article. Ecuador and Venezuela were not represented. Among the studies analyzed, the pie charts in Figure 4 shows that the most commonly used biomarker was muscle in animal tissues, and in studies conducted with human data, the most commonly used biomarker was hair, apart from blood and breast milk, each of which was presented in a single article.

**Figure 5:**
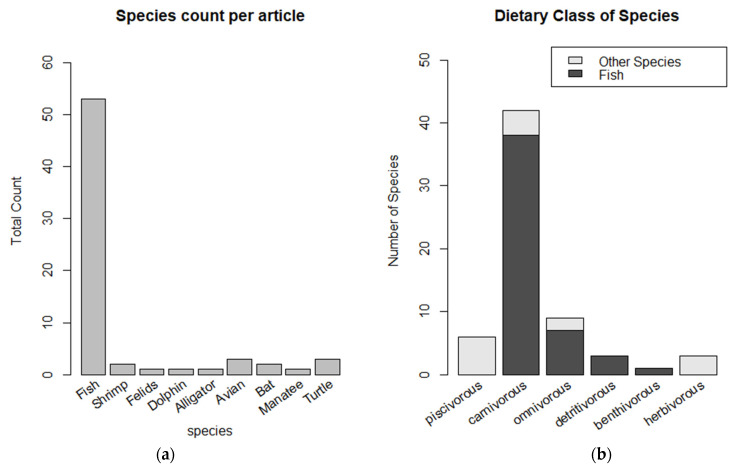
Main data sources obtained: (**a**) species and (**b**) trophic levels.

Among the studies that analyzed the presence of THg in animals, analyses of different fish species prevailed in addition to categorizing these feeding guilds (Figure 5). 

### 3.2. Overview of THg Levels in Animal Research

The results of these studies demonstrated that ecotoxicological research on Hg is predominantly conducted in aquatic ecosystems, mostly comprising fish species, considering exposure to indigenous and riverside communities in major rivers and tributaries. Therefore, to facilitate a more effective statistical overview, records that used fish muscle as a THg biomarker were reviewed separately. 

**Figure 6:**
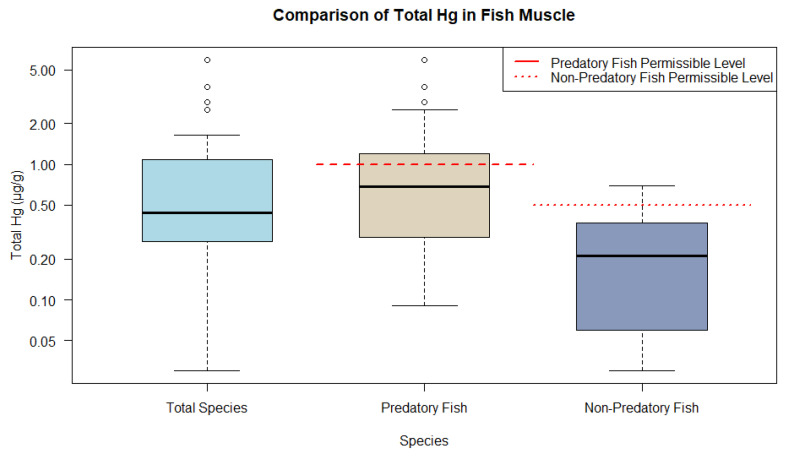
Representations of THg ranges in fish muscle tissue from animal research compared to WHO and MERCOSUR guidelines.

Accordingly, the predominance of fish muscle tissue could be plotted and categorized into predatory and non-predatory feeding guilds. The boxplots for fish indicate the distribution of THg into the following categories: all fish muscle (median 0.440 µg/g and interquartile range (IQR) 0.820 µg/g), predatory fish muscle (median 0.690 µg/g and IQR 0.900 µg/g), and non-predatory muscle (median 0.210 µg/g and IQR 0.300 µg/g). Predatory fish, classified as carnivores, exhibit THg ranges above the guidelines established by WHO, with levels being 1 µg/g and 0.5 µg/g, respectively, for predatory and non-predatory fish. In contrast, the non-predatory group, which included omnivores and other species, displayed significantly lower THg levels, suggesting a safe mean level (Figure 6). 

Accordingly, the highest frequencies of studies in the main tributaries of the Amazon River were for *Plagioscion squamosis Yesus*, *Serrasalmus rhombeus*, and *Cichla* spp. The carnivorous guild had the highest THg values (ranging from 0.086 to 5.920 μg/g), with a mean of 1.058 μg/g, followed by the omnivores (0.029 μg/g to 0.700 μg/g), with a mean of 0.316 μg/g, and the detritivores (0.064 μg/g to 0.370 μg/g), with a mean of 0.180 μg/g (Table 2). 

Additionally, studies evaluating fish in hydroelectric reservoirs and dam regions showed that Puruzinho and Catalão Lakes presented higher mercury concentrations of *S. rhombeus* (mean 0.789 μg/g, and range of 0.029 to 1.640 μg/g) [71,81,84]. On the other hand, the reservoirs of Belo Monte and Jirau Hydroelectric Plants showed high concentrations of mercury in *S. rhombeus* (0.480 μg/g) and lower concentrations in *Triportheus albus* (0.132 μg/g) [90,94].

Table 3 presents the remaining records, which either did not include fish muscle tissue analyzed for THg or were not sampled at Amazonian basin locations. These measured THg values include muscle tissue samples from fish along the Atlantic coast, hepatic tissue of fish, and tissues from species other than fish. These animal tissues show a mean THg value of 3.850 μg/g with a range from 0.011 to 17.900 μg/g, with the highest THg value being in the liver of *Arapaima gigas*. 

Variation from 0.010 to 17.900 µg/g of THg across ecoregions can be observed. Most sampling points were recorded in the southern Amazon forests, with a mean mercury level of 0.890 µg/g (range 0.016 to 11.570 µg/g), followed by the western Amazon forests, with a mean mercury level of 1.700 µg/g (range 0.150 to 17.420 µg/g). The least abundant points were recorded in the Guyana ecoregion, with a mean mercury level of 1.570 µg/g (range 0.165 to 5.920 µg/g).

**Figure 7:**
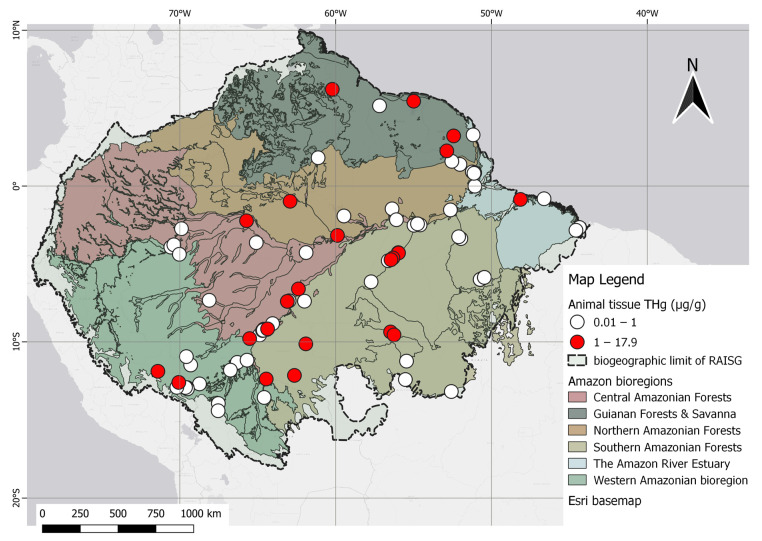
Spatial distribution of animal studies categorized by THg levels in fish in relation to the permissible reference concentration of 1.0 µg/g in the human diet, as established by WHO.

The Brazilian territory has the highest concentration of scientific reports, including the Amazon River and its major tributaries, with emphasis on the Madeira and Tapajós Rivers. Specifically, the Madeira River basin, which acts as a boundary between bioregions, has a mean mercury level of 0.470 µg/g in fish (range 0.014 to 4.046 µg/g). Also, Brazil Rondônia, near the region of Porto Velho, and the Tapajós River have a mean of 0.69 µg/g (range 0.0740 to 1.510 µg/g). Additionally, the Madre de Dios River basin in Peru has a mean of 0.720 µg/g of mercury (range 0.200 to 3.720 µg/g), while the mean mercury level in fish of the Amazon River was 0.673 µg/g (range 0.050 to 1.490 µg/g) (Figure 7). 

The highest levels of THg reported in the Amazon were in the skin tissues of *Panthera onca* (17.900 µg/g) and the hepatic tissue of *Arapaima gigas* (17.400 µg/g). In Brazil, the highest value of mercury in predatory fish was found in the black piranha (*S. rhombeus*), a carnivore, at 1.640 µg/g [84]. The highest muscle mercury concentration in countries outside Brazil was found in a study carried out at Oxbow Lakes, a natural protected area in Madre de Dios Province, Peru, at 3.170 µg/g, and along the Mazaruni River in Guyana, at 5.920 µg/g, both of which are areas close to mining activities [101,107]. 

### 3.3. Overview of THg Levels in Human Research

In a comparative analysis of indigenous populations (impacted or not by mining), and non-indigenous populations, this systematic review provided evidence that THg found in the hair of indigenous populations existed at marginally higher levels (median of 10.900 µg/g and IQR 4.900 µg/g) than that found among non-indigenous populations (median of 9.150 µg/g and IQR 7.490 µg/g). Studies that examined (small-scale) mining areas had a comparatively larger sampling range (IQR 10.652 µg/g) but a lower median (8.275 µg/g) within the studied population.

**Figure 8:**
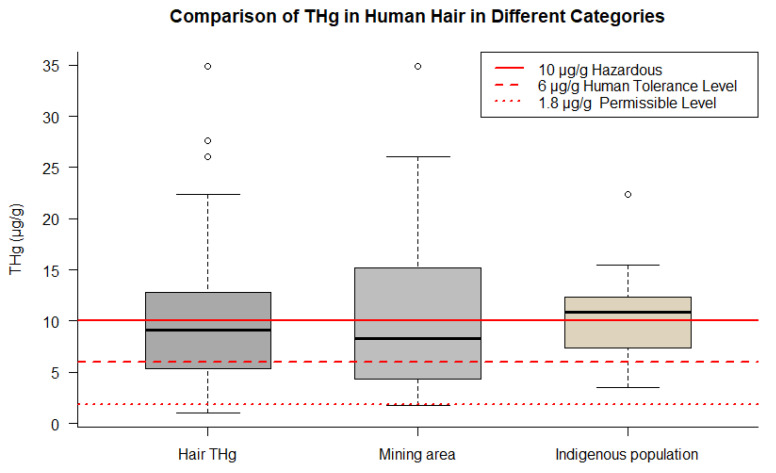
Comparison of THg concentrations in human hair across different groups. The figure points out data for overall samples, indigenous populations, and mining areas.

All categories analyzed exceeded the WHO guidelines for Hg in hair, suggesting that indigenous populations are exposed to higher levels of Hg, which may have implications for the health of these populations (Figure 8).

The total hair THg in humans ranged from 1.030 to 27.620 µg/g. Notably, we can distinguish the following groups: children, who had a mean of 8.81 µg/g (range 1.030 to 22.380 µg/g); indigenous populations, with a mean of 13.190 µg/g (range 2.060 to 34.900 µg/g); women of reproductive age, including mothers, pregnant women, and lactating women, who had a mean of 7.710 µg/g (range 2.120 to 12.80 µg/g); riverside populations, with mean concentrations of 10.610 µg/g (range 3.070 to 22.380 µg/g); and, finally, a mean of 11.930 µg/g (range 1.740 to 34.90 µg/g) reported for individuals living in mining areas. Table 4 shows a list of human studies with significant findings related to THg concentrations in human tissues.

Only two studies do not consider total human mercury concentrations in hair. One reported data from the breast milk (mean 0.010 µg/g THg) of lactating women to estimate exposure to neurotoxic metals influenced by small-scale mining in infants in Porto Velho, Rondônia, Brazil [154]. Another reported blood levels (mean 0.021 µg/g THg) in Amazonian juvenile populations in Cuniã, Brazil [155].

It should be noted that the indigenous community is well represented, especially in the Munduruku reserve/community in the Tapajós basin and in studies in the Madre de Dios region in Peru, as part of cohort studies [136,144,147] where comparisons were made between native demographics and mining hotspots, together with respective controls around the Amarakaeri Communal Reserve. Pregnant women, women of reproductive age, and juveniles received particular attention owing to their heightened vulnerability to mercury exposure. Interestingly, one study revealed that occupational Hg exposure from mining remained lower than levels observed in riverside communities, as exemplified by the riverside population of Itaituba (20 µg/g THg) compared to the mining region of Serra Pelada (1 µg/g THg) [146]. 

No statistical differences (*p* < 0.05) in THg concentration were found between the groups analyzed (children, indigenous, maternity, riverside, and those directly affected by mining) using MLG models. According to the summary in Table 5, no parameters were strongly correlated with concentrations in human hair. Logistic regression showed better a model fit than the Akaike Information Criterion (AIC), but all *p* values were > 0.05, i.e., no connection was demonstrated.

The spatial representation of human research is highest in the southern forest bioregion with 34 records. In this area, the highest density was recorded in the Tapajós basin, followed by western Amazon ecoregions with 28 records. The remaining studies were conducted in the other three regions.

**Figure 9:**
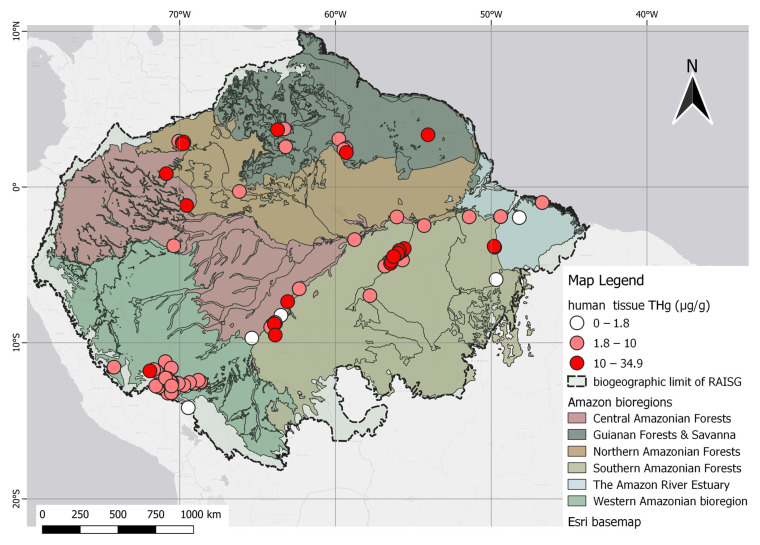
Spatial distribution of human studies according to THg levels, categorized as permissible (1.8 µg/g), dangerous (10 µg/g), and above the dangerous level (>10 µg/g).

The human data presented spatial trends nearly identical to studies concentrated in the Tapajós and Madeira River basins in Brazil and Madre de Dios in Peru. Evidence of three classes of THg gradients, along with several high-Hg points, can be visualized as deep red dots scattered across the map. This feature has also been observed in the northern and western areas of the Amazon Forest, including studies conducted in the state of Amazonas, Roraima in Brazil, and Nariño in the Colombian Amazon. These observations are illustrated in Figure 9.

### 3.4. Mining

Many studies have reported on Hg in regions with mining activities in floodplain areas, including all classes of Amazonian floodplains, grouped according to Dinerstein et al. [56]. It was possible to observe tributaries with intense mining activity near the sampling points, which are grouped on the map into classes according to distances, i.e., of 0–20 km, 20–50 km, and above 50 km, relative to mining polygons. The mining polygons (orange scheme) comprise a combination of datasets consisting of global mining footprints [58] with a layer of illegal mining, as adapted from RAISG [44].

Floodplain areas were specifically included in the analysis to represent zones of high water retention and the accumulation of mining tailings, which are factors that can contribute to Hg concentrations in the environment [84,156]. Notably, the extent of illegal mining is more widely recognized by initiatives such as RAISG, the data from which indicate an overlap of these activities with the studied areas. In particular, these illegal operations are prevalent in the western regions of the Napo River, an important tributary of the Amazon River basin, affecting the sample collection areas situated in the border zones that separate Peru, Colombia, and Brazil. Moreover, illegal mining activity is intense along the Madre de Dios River basin in Peru, with operations extending close to the designated sample collection areas. Certain sample points with close proximity classes (up to 20 km, and 20 km to 50 km) intersect with mining zones.

Highlights for animal studies from these points are in the Mazaruni River basin region in Guyana and the downstream Suriname River region, despite the low representation of study articles. Regions with significant research overlap and mining activity in the interior include the Tapajós basin, close to the Munduruku Reserve and Itaituba, and the Itacaiúnas River basin, situated between the Xingu and Tocantins River basins in Brazil. 

**Figure 10:**
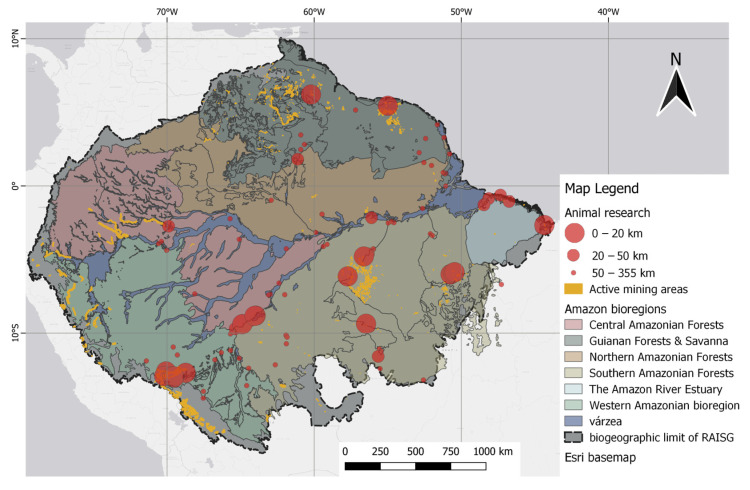
Spatial distribution of animal studies relative to mining polygons. Distance classes, as indicated by the 20 km and 50 km markers, were defined by the difference in point size.

Based on distance classification, mercury concentrations within a 20 km radius of the georeferenced mining sites (closest) had a mean of 0.870 µg/g (range 0.033–5.920 µg/g) for fish, wherein *Serrasalmus* spp. was the most frequently mentioned. The approximation category between 20 and 50 km had a mean of 0.506 µg/g (range 0.022 to 1.510 µg/g), and within this distance category, *Plagioscion squamosis Yesus* was the most cited fish species. Although higher, the mean muscle THg concentrations in fish closest to mining activity are still slightly below the 1 µg/g limit, indicating a permissible level for predatory fish, but it does exceed the permitted dietary reference of Hg set at 0.50 µg/g for their non-predatory counterparts. Figure 10 shows the point data for animal records, which are mapped alongside polygons that indicate small-scale mining. 

Research on human tissues has also been mapped along with the geospatial information of mining and indigenous territories. The results of human studies show that the highest number of studies were conducted close to the illegal mining polygons of RAISG in the Madre de Dios and Tapajós basins. The Madeira River basin is also the subject of a high number of studies, but with less illegal mining activity. This is because illegal mining follows the river flow, likely the result of alluvial mining activities. Consequently, the cities of Itaituba and Marabá, both in the Tapajós basin, are among the most frequent sample areas near illegal mining activities, reported in high numbers. 

Despite limited spatial representation in the north, some studies have highlighted the presence of mining activities. Among these, small-scale mining has been reported in the mountainous region of Marudi, which is located in Guyana [145]. Another point of interest is the Lawa River basin situated in French Guiana [8]. Additionally, the Yanomami indigenous region in Roraima, Brazil, which is in the Uraricoera River basin, is also mentioned [152]. Although limited in number, these studies hold significant value because of their proximity to mining areas in the Guiana Shield. 

**Figure 11:**
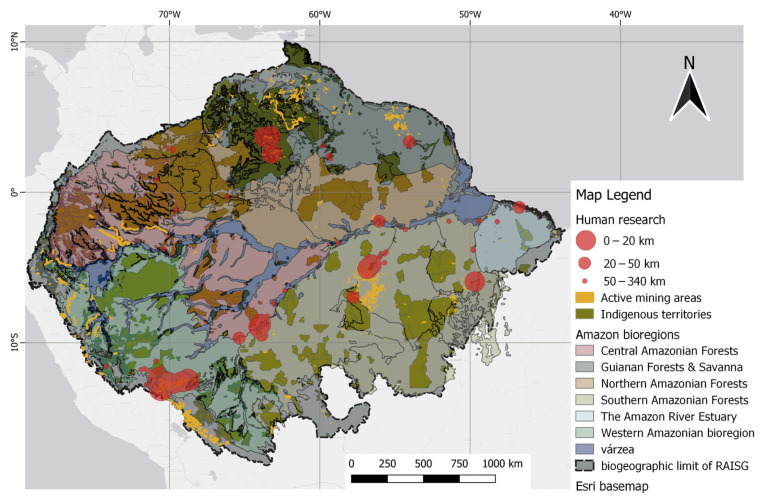
Spatial distribution of human studies relative to mining polygons. Distance classes, as indicated by the 20 km and 50 km markers, were defined by the difference in point size. Native/indigenous territories were included as darker overlays.

The human samples closest to mining areas up to 20 km had a mean THg of 4.301 µg/g (1 to 11.61 µg/g), while human samples at a distance of 20 km to 50 km had a mean of 6.782 µg/g (0.01 to 12.80 µg/g). Notably, all studies conducted within a 20 km radius of mining areas were also within a 17 km radius of indigenous territories. Figure 11 shows human studies related to mining polygons.

## 4. Discussion

This review provides insights into total Hg accumulated in animal and human tissues, as reported in recent surveys. The human records in this review overlap with those of the scoping review on neurotoxicity by Santos-Sacramento et al. [39] and confirm the trend of high representation of studies in Brazil.

This scoping review revealed a greater focus on the southwest region, particularly the basins of the Madre de Dios, Madeira, and Tapajós Rivers and their tributaries. The Madeira River and its floodplains serve as significant boundaries in the geographic zoning of the ecoregions considered in this study. The results related to animals were predominantly from aquatic ecosystems and were assessed within the context of the fish food web and bioconcentration of Hg, considering the differences between pelagic and demersal species and their distinct feeding groups in these tropical zones (feeding guilds) [41,77]. 

These studies partially cover the regions currently exploited for mining, but numerous regions are not represented or are under-represented in sampling locations for both humans and animals. The analysis of overall data in humans showed a median of alarmingly high levels of total Hg in hair (above 6 µg/g), thus exceeding the safe level for humans. This emphasizes the pressing necessity for comprehensive ecotoxicological studies in the Amazonian region. These studies form a basis for understanding the nature of mercury bioaccumulation and its spatial distribution in both animal and human tissues. High levels of Hg represent immediate environmental and public health concerns that require prompt attention.

We used a geographical dataset related to small-scale mining and its textual references in the reviewed items and created a spatial representation of the sample points. Based on this reference, it is noteworthy that certain areas, even those devoid of any mining activities, exhibit high mean tissue THg levels. This can be traced back to the cycling dynamics of Hg in the environment. Long-range deposits of Hg can be influenced by several factors. These include the transport of Hg through waterways, which leads to higher concentrations downstream owing to sedimentation, the atmospheric mercury (Hg) uptake by oxidation of Hg^0^ and Hg^2+^, and the role of larger aquatic animals. Aquatic animals contribute Hg through bioaccumulation and migratory behavior [61,157]. The migratory behavior of fish is crucial for spatial analysis, as they can be captured by fishermen far from critical mining contamination points or zones of high Hg content [65,147]. Lastly, the remobilization of Hg that persists in historic mining zones could currently impact the riverine populations that consume fish in abundance [142].

The well-studied tropical forest of the Amazon, which is an important terrestrial sink, mitigating the release of Hg into the atmosphere, could have its ability reduced by anthropogenically induced forest degradation [158]. 

In this review, both the volume of research conducted and the spatial distribution across the region are more extensively represented in animal studies compared to human studies. However, the high levels of Hg in human research call for equally distributed spatial representation, especially in indigenous communities where fish are a staple food. Even with diets primarily rich in fish, the total mercury levels in the indigenous populations showed lower variation [26].

Most human studies are based on hair as a non-intrusive and convenient matrix for detecting dietary Hg [54,159,160]. Interestingly, some studies note that proximity to mining is not necessarily associated with exposure risk [147]. However, despite the lower medians associated with higher levels of hair THg and greater variations in populations close to mining areas, the significant presence of Hg in these locations is undeniable. The same can be said for the observed overlap between fish habitats and floodplains, raising concerns about potential effects of nearby alluvial mining activity [161]. Notably, the presence of dredges in these areas further strengthens this suspicion [13,17,162,163]. 

High water levels generally present higher total Hg concentrations in fish tissues. Low water levels in blackwater tributaries, which are commonly believed to contain higher levels of inorganic Hg, do not necessarily result in higher tissue Hg, as low water factors may block the synthesis of Hg [77]. Occupational exposure from gold mining was unexpectedly lower than that in the riverside control group [146]. 

Hg release in the Amazon region remains ubiquitous and not clearly understood through both anthropogenic and natural pathways as a more elaborate combination of variables are required to correlate the impact to a point contamination, i.e., small-scale gold mining. The dietary habit and tissue Hg assessments derived in this review only reveal the after-effects and extent of Hg contamination within the region. The GLM and lower medians for THg in mining areas for humans suggest the possibility of elevated levels even in non-mining areas and non-rural communities (not riverine, tribal, or indigenous). 

While northern regions of the Amazon have a high occurrence of small-scale mining, research is noticeably lacking on both animals and humans in these regions.

Nonetheless, to address the mercury problem in the Amazon region, some directions and recommendations should be considered for future research and policy initiatives, as discussed below. 

The development and implementation of a regional Hg-monitoring network and database are essential. This would facilitate the collection, sharing, and dissemination of Hg data and information among different stakeholders and actors. It is important that this network includes all Amazonian nations with special attention given to those with fewer publications. The application and improvement of spatial analysis tools and techniques, such as the Geographic Information System (GIS), remote sensing, and spatial statistics, are essential to enhance the understanding and communication of spatial patterns and trends of mercury contamination and exposure in the region. The integration of RAISG geospatial datasets can be used as fundamental building blocks in this process.

The promotion and support of alternative and sustainable livelihoods and technologies in the artisanal and small-scale mining sectors are crucial. This would reduce the use and emission of mercury and improve the socioenvironmental conditions of miners and their communities. Establishing and enforcing regional and national standards and regulations for mercury management and control are required. This would harmonize and coordinate the efforts and actions of different countries and agencies to address the mercury problem in the region.

Based on the extent of research coverage of aquatic ecosystems, both in terms of fish muscle assessment and human consumption from fish-rich diets, a comprehensive meta-analysis could be proposed. This would statistically analyze the correlation between tissue Hg contamination and aqueous Hg concentrations in Amazonian hydrology and sediments, particularly in relation to the impact of clandestine small-scale mining. It would also consider Hg sedimentation known to be transported in aquatic environments, a phenomenon prevalent in the blackwater-flooded forests and floodplains of the Amazon region [34,164]. These environments carry suspended matter, which has been reported to contain high concentrations of Hg in these tributaries, increasing atmospheric concentrations owing to its volatility.

### Limitations

Some limitations of this study should be addressed. First, this review only focuses on tissue Hg and is thus limited by the lack of consideration of nearby sediment and water analyses. The search syntax and search engines used may have limitations by failing to include relevant articles. Importantly, assessing the contamination of ASGM by fish tissue Hg is less effective, as some argue, than measuring sediment Hg in local effluents [100]. The inclusion of alluvial sediment/water analysis, the consideration of downstream/upstream water flow relative to mining activities, and fish migratory factors could all have been factors accounting for lower fish muscle Hg levels near mining sites. 

Second, for georeferencing, only sampling locations geographically distinguishable on a regional scale were considered. The algorithm used for the global mining dataset was designed to detect disturbances in terrestrial or forested areas caused by mining activities. However, this approach resulted in limited data from RAISG on alluvial mining activities. 

Third, sample sizes were inconsistent among the studies with respect to THg data, as the number of fish per species varied significantly. The Hg concentration is often only reported for the entire collection, which may not accurately represent individual species or regions. Not all studies distinguished each sample region, often reporting the same averages (in mean or median) of geographically distant locations. The average Hg in these studies was indicated as either the mean or median, which, since they are different units of measurement, can affect the interpretation of overall data trends. The periods of sampling and tissue collection can vary drastically within the year of publication and are sometimes not indicated, leading to possible temporal bias. Some, but not all, authors indicated if they had cross-sectional or cohort studies. 

Fourth, different protocols and Hg reference materials were used to assess Hg in the tissues, such as Cold Vapor Atomic Absorption Spectroscopy (CVAAS), Cold Vapor Atomic Fluorescence Spectroscopy (CVAFS), and versions of Dogfish Muscle Certified Reference Material (DORM). Furthermore, the range of sampling periods, use of cohort versus cross-sectional studies, and consideration of wet weight versus dry weight, especially in fish tissues, may have contributed to variations in results. Wet weight was preferred based on its overall use in the studies reviewed.

Additionally, while some studies provide spatial details of sample points within their local study area, others do not, resulting in a map that may appear obscure owing to a lack of detailed information.

Lastly, only a few studies carried out a comprehensive data analysis, necessitating the expansion of the review period and, potentially, a systematic review or meta-analysis. These limitations highlight the need for more comprehensive and standardized research in this field.

## 5. Conclusions

Despite the wide range of studies on the presence of mercury in the Amazon, they are mainly concentrated in Brazil, in the western Amazon in the Madre de Dios region of Peru, and along the basins of the Tapajós River near Itaituba and the Madeira River near Porto Velho, Brazil. This coverage suggests a lack of studies assessing Hg contamination in other regions or countries of the Amazon, particularly Suriname, Ecuador, Venezuela, French Guiana, and English Guyana.

By broadening the scope of our systematic literature review, the results partially confirmed our initial hypothesis, in that we showed the highest concentrations of mercury in tissue samples collected near mining areas. This observation was particularly evident in studies that specifically evaluated the impacts of mining. It was noted that indigenous populations were more susceptible to mercury exposure, raising concerns discussed in the review. Interestingly, even areas located up to 50 km from mining activities exhibited higher mean THg levels in human hair, surpassing the 6 µg/g threshold established by the WHO. This suggests that the impact of mining activities on mercury levels extends well beyond the immediate vicinity of the mines.

Our review highlights that mining activities are a significant source of downstream Hg contamination and bioaccumulation in aquatic ecosystems. Understanding both the progression of mercury contamination by mining and the distribution and dispersion of downstream mercury concentrations is critical for properly assessing its effects on animals and humans. It is also important to highlight the high concentrations of mercury in predatory fish, as well as the potential health risks to human populations that rely heavily on fish as a staple in their diet. This finding underscores the need for stringent regulations and effective mitigation strategies for mining practices. Additionally, as anticipated in some studies, the bioaccumulation of mercury in fish demands that their migratory behavior be considered since fish can reach areas not known for mining activities. 

This review documents a significant impact of mercury contamination on human and animal populations in the Amazon region and identifies gaps in geographic coverage. It is anticipated that these findings will lead to more comprehensive studies, particularly in areas that have not been adequately studied in previous research.

## Figures and Tables

**Table 1:** Search syntax (CH3Hg^+^ also termed as MeHg, asterisk (*) used as wildcard operator).

Arguments	Search Syntax
mercury in the Amazon	Amazon * AND (mercury * OR hg OR MeHg OR methyl * OR “total mercury”)
activity	AND (mining OR artisanal OR asgm OR “gold mining *” OR garimp * OR alluvial OR contamination OR “inorganic contaminant *” OR “trace mineral *” OR trophic)
specimen	AND (fish OR species OR population OR aquatic OR bioaccumulation OR community OR riverine OR “human hair” OR indigenous OR human OR nonhuman OR animal)
conditions	AND (exposure OR accumulation OR concentration OR content OR consumption)
biomarker	AND (tissue OR muscle OR protein OR hair OR blood)
exclude ex situ	NOT (farm OR agriculture)

**Table 2:** Highest mean or median THg values reported in the muscle records of fish grouped by basin location with indication of fish feeding habits and whether mining activities were part of the study.

Hydrographic Basin *	Mentioned Mining?	Country	Study	Species/Feeding Guild	THg (μg/g) Based on Mean/Median ^†^
A	No	Brazil	[64,65] **	*Hoplias malabaricus*, carnivore	0.947 ^†^
A	No	Brazil	[66]	*Carcharhinus acronotus*, carnivore	1.120
A, T	Yes	Brazil	[67]	*Arapaima* sp., carnivore	0.375
A, T	No	Brazil	[68]	*Plagioscion squamosisYesus*, carnivore	1.510
A	Yes	Brazil	[69]	*Pseudoplatystoma tigrinum*, carnivore	0.920
A	No	Brazil	[70]	*Ageneiosus inermis*, carnivore	0.691
A	No	Brazil	[71]	*Acestrorhyncus falcirostris*, carnivore	1.490
A	No	Brazil	[72]	*Colomesus asellus*, omnivore	0.350
T	Yes	Brazil	[73]	Carnivorous group: *Cichla monoculus*, *Plagioscion squamosis Yesus*, and *Serrasalmus calmoni*	0.668
T	Yes	Brazil	[74]	*Plagioscion squamosis Yesus*, carnivore	0.730
T	Yes	Brazil	[75]	*Cichla pinima*, carnivore	1.172
T	No	Brazil	[76]	*Serrasalmus rhombeus*, carnivore	0.088
M	No	Brazil	[77]	Carnivorous group: *Plasgioscion squamosis Yesus*, *Calophysus macropterus*, *Cichla pleiozona*, and *Hoplias malabaricus*	0.970
M	No	Brazil	[78]	*Prochilodus nigricans*, detritivore	0.064
M	No	Brazil	[79]	*Serrasalmus rhombeus*, carnivore	0.263
M	Yes	Brazil	[80]	*Triportheus angulatus*, omnivore	0.290
M	No	Brazil	[81]	*Triportheus albus*, omnivore	0.029
M	No	Brazil	[82]	*Pinirampus pirinampu*, benthivore	0.060
M	No	Brazil	[83]	*Arapaima gigas*, carnivore	0.153
M	No	Brazil	[84]	*Serrasalmus rhombeus*, carnivore	1.640
M	No	Brazil	[85]	*Plagioscion squamosisYesus*, carnivore and *Colossoma macropomum*, omnivore	0.086
M	No	Brazil	[86]	*Brachyplatystoma filamentosum*, carnivore	0.402
M	No	Brazil	[87]	*Calophysus maropterus*, carnivore	1.400
M	No	Peru/Brazil (Border)	[88]	*Calophysus maropterus*, carnivore	0.229
M	No	Brazil	[89]	*Cichla* spp., carnivore	0.128
M	No	Brazil	[90]	*Semaprochilodus* spp. (Jaraqui), detritivore	0.132
M	No	Brazil	[91]	*Serrasalmus rhombeus*, carnivore	0.268
M	No	Brazil	[92]	*Cichla* spp. (Tucunaré), carnivore	0.435
M	Yes	Brazil	[93]	*Serrasalmus rhombeus*, carnivore	0.417
X	No	Brazil	[94]	*Hemiodus unimaculatus*, omnivore	0.480
Tp	No	Brazil	[95]	*Serrasalmus rhombeus*, carnivore	0.304
Tp	No	Brazil	[96]	*Brycon falcatus*, omnivore	0.052
Ar	Yes	Brazil	[12]	Carnivorous group: *Ageneiosus inermis*, *Boulengerella cuvieri*, *Cichla monoculus*, and *Hoplias aimara*	0.580
Ar	Yes	Brazil	[97]	*Curimata incompta*, detritivore	0.370
Ar	Yes	Brazil	[98]	*Plagioscion squamosis Yesus*, carnivore	0.320 ^†^
J	No	Brazil	[99]	*Plagioscion squamosis Yesus*, carnivore	1.090
MD	Yes	Peru	[100]	*Serrasalmus* spp., carnivore	0.280
MD	Yes	Peru	[101]	*Serrasalmus* spp., carnivore	3.720
B	No	Bolivia	[102]	*Brycon amazonicus*, omnivore	0.700
Br	Yes	Brazil	[103]	*Pinirampus pirinampu*, carnivore	0.869
Br	Yes	Brazil	[32]	*Pygocentrus nattereri*, carnivore	1.215
M	Yes	Brazil	[104]	*Serrasalmus rhombeus*, carnivore	0.283
Su	Yes	Suriname	[105]	Multiple, high THg in carnivores: *Acestrorhynchus microlepsis*, *Hoplias malabaricus*, *Cichla ocellaris*, *Serrasalmus rhombeus*, and *Pristobrycon eigenmanni*	2.528
C	Yes	French Guiana	[106]	*Hoplias aimara* and *Boulengerella cuvieri*, carnivore	2.900
Mz	Yes	Guyana	[107]	*Ageneiosus ucayalensis*, carnivore	5.920

* Main Amazon River: A, Tapajós River: T, Madeira River: M, Xingu River: X, Teles Pirés River: Tp, Jiparaná River: J, Madre de Dios River; MD, Beni River: B, Rio Branco: Br, Suriname River: Su, Camopi River: C, Mazaruni River: Mz. ** These studies used the same fish muscle tissue collection and mean THg. † Records that only provided median THg.

**Table 3:** Tissue THg in fish, birds, mammals, and crustaceans according to location in the Amazon region and mining area coverage.

Location	Mentioned Mining?	Study	Species	Tissue	Mean THg (μg/g)
Madeira River, Rondônia, Brazil	No	[108]	*Arapaima gigas*, fish, carnivore	hepatic	17.420
Araguari River, Amapá, Brazil	No	[109]	*Anodus orinocensis*, fish, omnivore	hepatic	0.500
Madeira River, Brazil	No	[110]	*Macrobrachium amazonicum*, shrimp, omnivore	muscle	0.610
Atlantic Coast, Ilha dos Caranguejos, Brazil	No	[111]	*Sciades herzbergii*, fish, omnivore	muscle	0.033
Mamirauá, Amazonas, Brazil	No	[112]	*Panthera onca*, mammal, carnivore	pelage	17.900
Guaporé River, Brazil	No	[113]	*Inia boliviensis*, mammal, piscivore	adipose	1.323
Beni River, Bolívia	No	[114]	*Caiman yacare*, reptile, piscivore	muscle	0.150
Madeira River, Brazil	No	[115]	*Ardea cocoi*, bird, carnivore	feather	4.046
Biological Station Cocha Cashu, Peru	No	[116]	*Rhynconycteris naso*, mammal, carnivore	pelage	7.440
Madre de Dios, Peru	Yes	[117]	*Phyllostomus elongatus*, mammal, carnivore	pelage	0.660
Figueiredo, Amazonas, Brazil	No	[118]	*Trichechus inunguis*, mammal, herbivore	muscle	0.059
Arauca River and Orinoco River, Colombia	Yes	[119]	*Inia* sp. and *Sotalia* sp., mammal, piscivore	muscle	0.870
Itapuru mirim Lagoon, Brazil	No	[120]	*Podocnemis unifilis*, reptile, herbivore	muscle	0.011
Xingu and Teles Pires’ Rivers, Brazil	No	[121]	*Podocnemis unifilis*, reptile, herbivore	muscle	0.134
Uatumã River, Balbina Brazil	No	[122]	*Podocnemis expansa*, reptile, omnivore	muscle	0.109
Teles Pires, Brazil	No	[123]	*Chloroceryle amazona*, bird, piscivore	feather	11.570
Teles Pires, Brazil	No	[124]	*Chloroceryle amazona*, bird, piscivore	feather	4.000
Madeira River, Brazil	No	[125]	*Macrobrachium depres Yesanum*, *Macrobrachium jelskii*, shrimp, omnivore	muscle	0.022

**Table 4:** Total THg in human hair of Amazonian populations.

Hydrographic Basin *	Mining Mentioned?	Country	Study	Community/Population	THg (μg/g) Based on Mean/Median ^†^
To	Yes	Brazil	[126]	Adults (18–70), fish as a staple food, near the reservoir of Tucuruí Dam.	10.900
U	Yes	Brazil	[8]	Age groups and comparison of various villages. Yanomami indigenous reserve, with a high diet of fish and nearby mining activity.	15.500 ^†^
M	Yes	Brazil	[127]	Age groups of adults ranging from 17 to 92 years.	26.030
To, Ta	Yes	Brazil	[128]	Adult riverside dwellers only (18 to 60 years old).	4.500
Ta	Yes	Brazil	[129]	Riverside dwellers only; adult women (13 to 53 years old).	9.150
Ta	Yes	Brazil	[130]	Munduruku Indigenous Reserve. Comparison between villages.	7.400
M, Ta	No	Brazil	[74]	Pregnant women (18 to 40 years old). Fish Diet.	6.070
MD	Yes	Peru	[62]	Urban and rural demographic comparison with a focus on fish diets.	1.740
A	Yes	Colombia	[69]	Indigenous community in Puerto Nariño. Mean age~35 years. Diet rich in fish.	5.310
Ta	No	Brazil	[11]	Munduruku Indigenous Reserve. Comparison between villages and age categorization with juvenile, childbearing-age, and other adults.	11.500
Ta	No	Brazil	[131]	Munduruku Indigenous Reserve. Comparison between villages and the exclusively juvenile population.	11.800
Ta	No	Brazil	[10]	Munduruku Indigenous Reserve. Comparison between villages. Ages > 12 years.	7.400
A, Cg, Ta	Yes	Brazil	[132]	Youth and adults in riverside communities.	12.700
MD	Yes	Peru	[133]	Matsigenka Indigenous community (ages 1 to 65).	11.830
MD	No	Peru	[134]	Riverside communities. High-fish diet.	4.800
MD	No	Peru	[135]	Comparison of various dwellings in the Amarakaeri Reserve, age categorization (under 5 years old, and 5 to 11 years old).	1.030 ^†^
MD	Yes	Peru	[136]	Comparison of various dwellings in the Amarakaeri Reserve.	4.150
A	Yes	Brazil	[137]	Riverside population. Prenatal exposure, women of childbearing age (15 to 49 years)	6.490
To, Ta	No	Brazil	[138]	Riverside communities with ages between 19 and 70 years (high THg).	15.900 ^†^
M, N	Yes	Brazil	[139]	Lactating women.	2.120
To	No	Brazil	[140]	Riverine populations in the Tucuruí Dam reservoir area.	8.120 ^†^
CP	Yes	Colombia	[26]	Indigenous communities in Tarapacá village.	17.800 ^†^
MD	Yes	Peru	[141]	Women of childbearing age.	5.500
Ta, To	No	Brazil	[142]	Children from riverside villages; born to women aged between 25 and 40. Fish-rich diet and primary exposure to Hg.	22.380
M	No	Brazil	[143]	Children/adolescents aged 6 to 14 along the Madeira River.	3.070
MD	Yes	Peru	[144]	Comparison of various dwellings in the Amarakaeri Reserve. Indigenous Native children (6 to 15 years old)	2.060
RK	Yes	Guyana	[145]	Indigenous people from the Rupununi region (15 to 78 years old). High-fish diet. Comparison of mining area and control area.	27.620
Ta, To	Yes	Brazil	[146]	Riverine men and miners and the THg among them. High-fish diet. Itaituba and Serra Pelada.	20.000
MD	Yes	Peru	[147]	Comparison of various dwellings in the Amarakaeri Reserve. Women of childbearing age (15 to 49 years).	3.500 ^†^
M	Yes	Brazil	[148]	Riverine, rural, mining, and urban communities. Women of childbearing age.	12.220 ^†^
To	No	Brazil	[149]	Adults (18–70 years), Riverine populations in the Tucuruí Dam reservoir area.	7.900 ^†^
Ap	Yes	Colombia	[150]	Population in different locations in mining regions. High-fish diet.	14.920
Ta	No	Brazil	[151]	Munduruku indigenous reserve. Comparison between villages and categorization. Diet rich in fish. Ages over 12 years old.	8.500
Ap	Yes	Colombia	[14]	Indigenous population of the Yaigojé Apaporis National Natural Park.	34.900
Ta	No	Brazil	[53]	Adult riverside residents only (18 to 60 years old). High consumption of fish.	10.800
Co	Yes	French Guiana	[152]	Only pregnant women, ethnic groups considered (15 to 41 years old); tribal and indigenous communities of Wayana.	12.800
M	No	Brazil	[153]	Mothers and children in childbirth and after pregnancy at 6, 24, and 59 months of age.	11.610

* Madre de Dios River: MD, Tapajós River: Ta, Cotuhe and Putumayo Rivers: CP, Campina Grande River: Cg, Tocantins River: To, Apapóris River: Ap, Uiaiacás River: U, Courantyne River: Co, Rupununi and Kuyuwini Rivers (Guyana): RK. † Records that only provided median THg.

**Table 5:** General Linear Model (GLM) and logistic regression of the effect of THg on mining-related parameters and demographic classes in human studies.

		GLM	Logistic Regression
	AIC	257.26	53.867
	Parameters	*p*-Value	*p*-Value
THg	Children	0.6055	0.416
	Indigenous	0.4201	0.273
	Maternity	0.2364	0.661
	Riverside	0.6108	0.287
	Mining	0.2413	0.774
